# AI-driven remote sensing enhances Mediterranean seagrass monitoring and conservation to combat climate change and anthropogenic impacts

**DOI:** 10.1038/s41598-024-59091-7

**Published:** 2024-04-10

**Authors:** Masuma Chowdhury, Alejo Martínez-Sansigre, Maruška Mole, Eduardo Alonso-Peleato, Nadiia Basos, Jose Manuel Blanco, Maria Ramirez-Nicolas, Isabel Caballero, Ignacio de la Calle

**Affiliations:** 1 Quasar Science Resources, S. L. Camino de las Ceudas 2, 28232 Las Rozas de Madrid, Madrid Spain; 2 Instituto de Ciencias Marinas de Andalucía (ICMAN), Consejo Superior de Investigaciones Científicas (CSIC), Avenida República Saharaui, 11510 Cadiz, Spain; 3 Departamento de Física Aplicada, Instituto Universitario de Investigación Marina (INMAR), Universidad de Cádiz, Campus de Excelencia Internacional/Global del Mar (CEI·MAR), Puerto Real, Cadiz Spain

**Keywords:** Environmental impact, Marine biology

## Abstract

Seagrasses are undergoing widespread loss due to anthropogenic pressure and climate change. Since 1960, the Mediterranean seascape lost 13–50% of the areal extent of its dominant and endemic seagrass-*Posidonia oceanica*, which regulates its ecosystem. Many conservation and restoration projects failed due to poor site selection and lack of long-term monitoring. Here, we present a fast and efficient operational approach based on a deep-learning artificial intelligence model using Sentinel-2 data to map the spatial extent of the meadows, enabling short and long-term monitoring, and identifying the impacts of natural and human-induced stressors and changes at different timescales. We apply ACOLITE atmospheric correction to the satellite data and use the output to train the model along with the ancillary data and therefore, map the extent of the meadows. We apply noise-removing filters to enhance the map quality. We obtain 74–92% of overall accuracy, 72–91% of user’s accuracy, and 81–92% of producer’s accuracy, where high accuracies are observed at 0–25 m depth. Our model is easily adaptable to other regions and can produce maps in in-situ data-scarce regions, providing a first-hand overview. Our approach can be a support to the Mediterranean *Posidonia* Network, which brings together different stakeholders such as authorities, scientists, international environmental organizations, professionals including yachting agents and marinas from the Mediterranean countries to protect all *P. oceanica* meadows in the Mediterranean Sea by 2030 and increase each country’s capability to protect these meadows by providing accurate and up-to-date maps to prevent its future degradation.

## Introduction

Seagrasses, the submersed marine flowering plants, are undergoing substantial decline due to direct and indirect anthropogenic activities and climate change. The recent publication^1^ estimated the global seagrass area to date as 160,387 to 266,562 square km, which is significantly lower than the previously reported global seagrass spatial distribution ranging from 177,000 to 600,000 square km^2,3^. Their loss rates accelerate from 0.9% year^−1^ in the 1940s to 7% year^−1^ toward the end of the twentieth century, making them one of the most threatened ecosystems on Earth^4,5^. Since 1960, the Mediterranean seascape lost 13–50% of the areal extent of its most common and endemic *Posidonia oceanica* seagrass meadows^6^. If the recent heat waves in the Mediterranean Sea continue, this seagrass may disappear within 100–150 years^7^. Recently, efforts to protect *P. oceanica* have led to the creation of the *Mediterranean Posidonia Network *(*MPN*, https://medposidonianetwork.com/) including more than 50 stakeholders from 10 countries, which seeks to effectively protect 100% of *P. oceanica* by 2030 by introducing innovative tools and raising awareness at the local, national, and international levels about the importance of this species^8^. However, the lack of a multi-temporal monitoring system over large areas, and identifying the hotspots to mitigate the rapid degradation caused by anthropogenic pressures are becoming a major concern for Mediterranean coastal managers, affecting the conservation program to protect this species.

**Figure 1:**
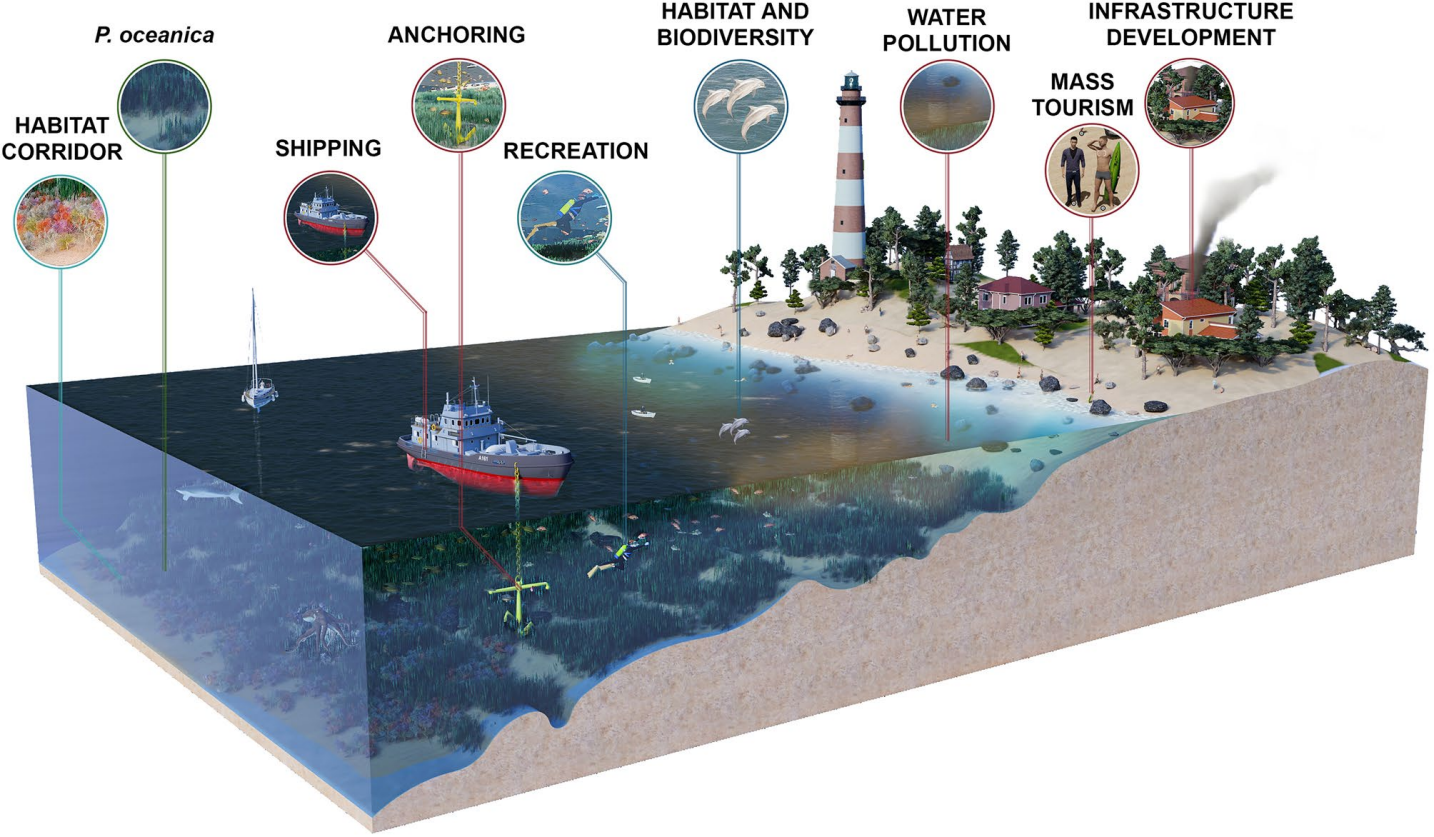
Why conservation of *P. oceanica* is important?. Conceptual diagram depicting the ecosystem services of *P. oceanica* (outlined by blue lines) and the anthropogenic pressures on the meadows (outlined by red lines). The meadows are endemic and dominant seagrass species in the Mediterranean Sea that controls its coastal and aquatic ecosystem. They provide shelter as well as act as a nursery for thousands of marine animals and plants, several of which are endangered species such as sea turtles and dugongs. The plant itself oxygenates the water and stabilizes the sandy shores and sea beds. They provide food directly through grazing or indirectly through detritus cycle^9^. They create ecological corridors between different habitats and are also globally significant carbon sinks^13^. The meadows are threatened by different anthropogenic activities, i.e. coastal infrastructure development, water pollution, fishing, shipping and anchoring. Over the last 50 years, the Mediterranean basin lost 13–50% of the areal extent of this meadow^4^. The recent warming of the Mediterranean Sea exaggerates the situation even more, which calls for continuous monitoring of the meadows, identifying potential areas to mitigate the human impacts as well as selection of sites for conservation and restoration. Sketchup 2022 (https://www.sketchup.com/offline-download) and Photoshop 2023 (https://www.adobe.com/products/photoshop.html).

*Posidonia oceanica* covers 50,000 square km of coastal sandy and rocky areas consisting of 25% of the Mediterranean Sea bottom at 0–40 m depth^9,10^. The meadows are home to about 20% of Mediterranean sea life species and can accommodate up to 350 animal species per hectare^11–13^. They contribute to different coastal processes like sediment deposition, and attenuate currents and wave energy^14^. They are globally significant carbon sinks with greater organic carbon density compared to estuarine mangroves, peatlands and tropical forests^15,16^, thus playing a key role in climate change mitigation^17^. However, the meadows are susceptible to increasing heat waves and climate change^18–20^. Besides, the meadows are under substantial threat by different anthropogenic activities, i.e., 22% of construction of coastal infrastructure, 23% of water pollution, 14% of invasive species, 18% of Fishing, 5% of shipping and 18% of modifications of marine currents and hydrography^21^ (Fig. 1). These threats, coupled with the slow horizontal growth of *P. oceanica* (1–6 cm/year), underscore the critical importance of preserving the meadows in their original state^22^. The European Union (EU) Habitats Directive 92/43/EEC has listed *P. oceanica* meadows as a priority habitat. *P. oceanica* is also a protected species in the Natura2000 networks within the EU because of its significant influences on the surrounding biological, biogeochemical and physical processes in the littoral^23^.

A successful conservation programme for *P. oceanica* involves knowledge about the spatial extent of the meadows, understanding processes of degradation^24^; identifying causes of degradation^25^; designing and implementing effective monitoring programmes to manage, restore, or create these seagrass meadows^26,27^. This demands fast, cost-effective and validated methods to observe and monitor the present state, spatial pattern, and dynamics at appropriate spatial and temporal scales^28,29^. Multiple studies^30–39^ presented the potential of remote sensing data in mapping the extent of this seagrass species, complementing the traditional in-situ monitoring system. However, they were mostly for proof of concept to utilize satellite-derived data and were hardly capable of supporting the continuous monitoring system since they are computationally expensive, and require validated methods that can map across large areas with adequate accuracy and precision.

**Figure 2:**
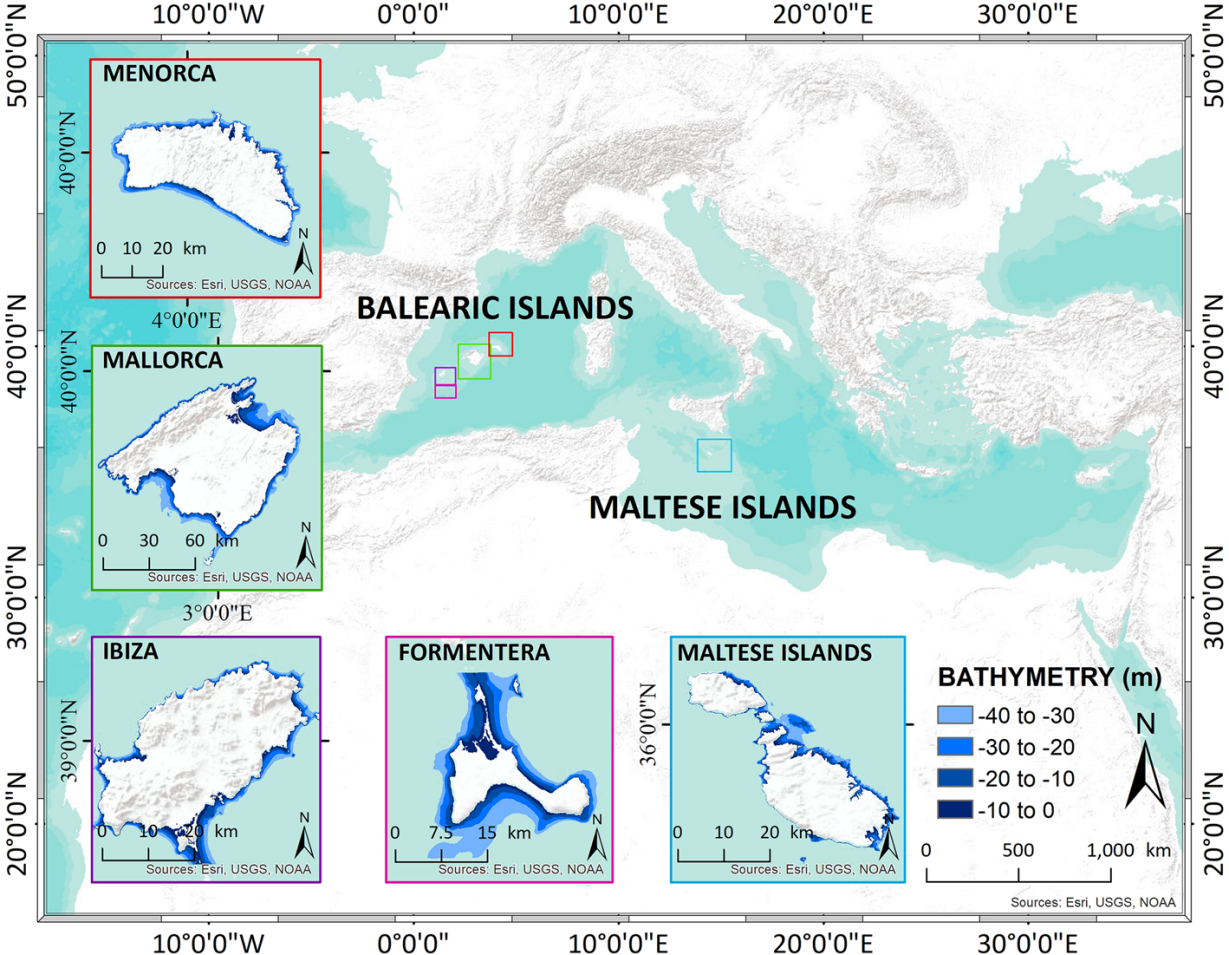
Study regions. The study regions include four Spanish Islands, namely Menorca, Mallorca, Ibiza and Formentera from the Balearic Islands located in the western Mediterranean Sea, and the Maltese Islands located in the central Mediterranean Sea. The continental shelf of the Balearic Islands is nearly horizontal and situated at 93 m depth on an average. The coastal waters are highly transparent due to the absence of rivers and the infiltration of rainfall in the karstic soils. Currently, 73% of the Balearic coastline is under protection through the Natural Areas of Special Interest, National Natural Park, Natura2000 sites, and the UNESCO Biosphere Reserve. In contrast, the Maltese Islands consist of shallow water, and the surrounding seafloor comprises an irregularly shaped continental shelf. Sandy bottoms with *P. oceanica* and *C. nodosa* characterize the dominant benthic habitats in shallow waters, mainly the offshore east coast of the Maltese archipelago. Python 3.7 (https://www.python.org/downloads/release/python-370/) and ArcGIS 10.5 (https://desktop.arcgis.com/en/arcmap/10.5/get-started/installation-guide/installing-on-your-computer.htm?).

With growing archives of freely accessible earth observation data of adequate spatial and temporal resolution, along with fast-growing computational processing capabilities, we now have the potential tools to support mapping and monitoring of *P. oceanica* over large regions. To our knowledge, there is no use of remote sensing techniques coupled with deep-learning-based artificial intelligence, integrated within a pipeline technique that facilitates reproducibility and scalability to monitor the multi-temporal spatial extent of this seagrass species, thus supporting the conservation activity. In this study, we present an operational approach aiming to fill this gap. Our approach corresponds to a Scientific Exploitation Platform (SEP) dedicated to the extraction and processing of Earth observation (EO) products by adding one more layer on top of the existing tools to take the exploitation of the raw satellite data one step further. The proposed pipeline used Sentinel-2 data for the artificial intelligence deep-learning neural network (DLNN) to produce multi-temporal maps of *P. oceanica*. Here, we present the results for four major islands of the Balearic Islands, i.e. Menorca, Mallorca, Ibiza, and Formentera in Spain, and the Maltese Islands (Fig. 2). For these regions, we have trained the DLNN model and evaluated its performance under the scheme of long-term monitoring and evaluation to support conservation and ecosystem management to minimize climate change and anthropogenic impacts. This study should be of interest to MPN as well as the scientists, conservationists and/or coastal managers, who are responsible for the challenging short-term management and long-term policy decisions to protect this type of natural resources.

## Results and discussion

### Maps of *P. oceanica*

**Figure 3:**
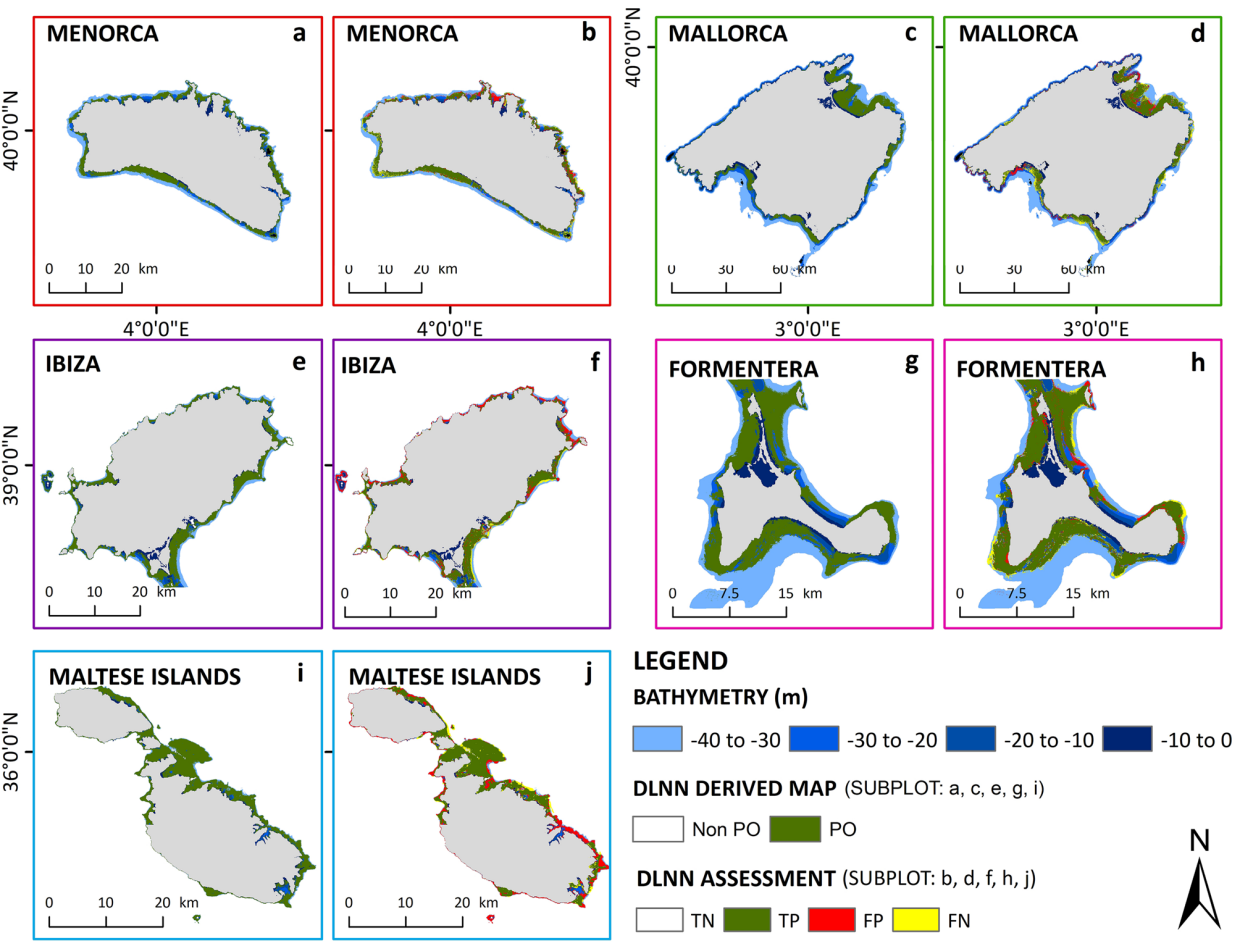
Regional maps of *P. oceanica* meadows. Regional maps of *P. oceanica* obtained through training the DLNN model with the corresponding in-situ data for the Balearic and Maltese Islands. The maps have 10 m spatial resolution. Each pixel was identified as either PO or Non PO by the DLNN model. Subplots (**a**,**c**,**e**,**g**,**i**) demonstrate the spatial extent of the meadows identified by the DLNN model in Menorca, Mallorca, Ibiza, Formentera, and the Maltese Islands, respectively. Subplots (**b**,**d**,**f**,**h**,**j**) demonstrate model’s performance per pixel for the corresponding regions. TN is True Negatives (pixels correctly identified as Non-PO), TP is True Positives (pixels correctly identified as PO), FP is False Positives (Non-PO pixels classified as PO), and FN is False Negatives (PO pixels classified as Non-PO). The land is shown in grey colour. Python 3.7 (https://www.python.org/downloads/release/python-370/) and ArcGIS 10.5 (https://desktop.arcgis.com/en/arcmap/10.5/get-started/installation-guide/installing-on-your-computer.htm?).

We have detected 577.29 and 74.27 square km of *P. oceanica* respectively in the Balearic Islands and the Maltese Islands during 2021 (Fig. 3). Our proposed method is based on pixel-by-pixel classification schemes, and the final output map has 10 m spatial resolution. We have stacked 3–9 atmospherically corrected clear Sentinel-2 level-2 images per tile (Supplementary Table S1) and applied the DLNN model with the corresponding in-situ and bathymetry data. The yearly stacked image using the median value for each pixel across the considered images reduced the potential misclassification due to non-persistent and seasonal variations in algal growth that may have similar signals. It was evident that depending on the region, our proposed approach was capable of mapping *P. oceanica* with 74–92% of overall accuracy, 72–91% of user’s accuracy, and 81–92% producer’s accuracy (Table 1). When a satellite pixel contained a region with sparsely distributed or unhealthy *P. oceanica* (according to the in-situ data), it was classified as false negative (FN) due to weak spectral signal. In contrast, when majority parts of the pixel contained healthy *P. oceanica*, it was classified as false positives (FP) due to strong spectral signal. However, in the north and northeast parts of Ibiza, we have observed swell noise in all the available Sentinel-2 images that caused residual noise in the yearly stacked image and resulted in misclassification. In contrast, we have noticed a different scenario in Malta. In the southeastern part of Malta, we observed the presence of spectral signals coming through the water column, which were identified by the DLNN model as *P. oceanica*. In this context, there could be two possible explanations, i.e., incomplete in-situ database, where the identified patches of *P. oceanica* were not included; consequently, it was depicted as FP during accuracy assessment; or, the presence of another seagrass species with similar signals. In case of the second one, a more detailed spectral inspection of other visible and near-infrared bands along with the RGB bands could be a solution to distinguish different habitats, which will be a topic of future studies. However, it should be noted that the in-situ data used in this study came from several years of field campaigns and might not be up-to-date in every region. As a result, in some places, the FP and FN identified in the final maps could be a true presentation of the current scenario and stressed the need for further investigation.

**Table 1:** DLNN model performance in regional mapping of *P. oceanica* during 2021.

Location	Overall accuracy (%)	User’s accuracy (%)	Producer’s accuracy (%)
Formentera	92	91	91
Ibiza	78	72	88
Mallorca	88	84	81
Menorca	86	83	86
Maltese Island	74	74	92

Our model obtained a higher user’s and producer’s accuracy at 0–25 m depth (Table 2), which was the most representative depth limit of the seabed detection using Sentinel-2 satellite in the Mediterranean Sea^37,40^. As the water was transparent and Sentinel-2 could see accurately enough, the model showed higher accuracy at this depth. With increasing depth and light attenuation, there could still be some *P. oceanica* living on the sea bottom up to 40 m depth^41,42^ but the satellite could not see them accurately enough. This explained the model’s performance with comparatively low accuracies at that depth.

**Table 2:** DLNN model performance by depth in regional maps of *P. oceanica* during 2021.

Statistics	Depth									
	Menorca		Mallorca		Ibiza		Formentera		Maltese Islands	
	0–25 m	25–40 m	0–25 m	25–40 m	0–25 m	25–40 m	0–25 m	25–40 m	0–25 m	25–40 m
Overall accuracy (%)	86	86	86	90	85	70	93	90	77	71
User’s accuracy (%)	86	79	85	81	81	56	93	88	77	71
Producer’s accuracy (%)	90	82	86	69	95	76	96	83	92	92

**Figure 4:**
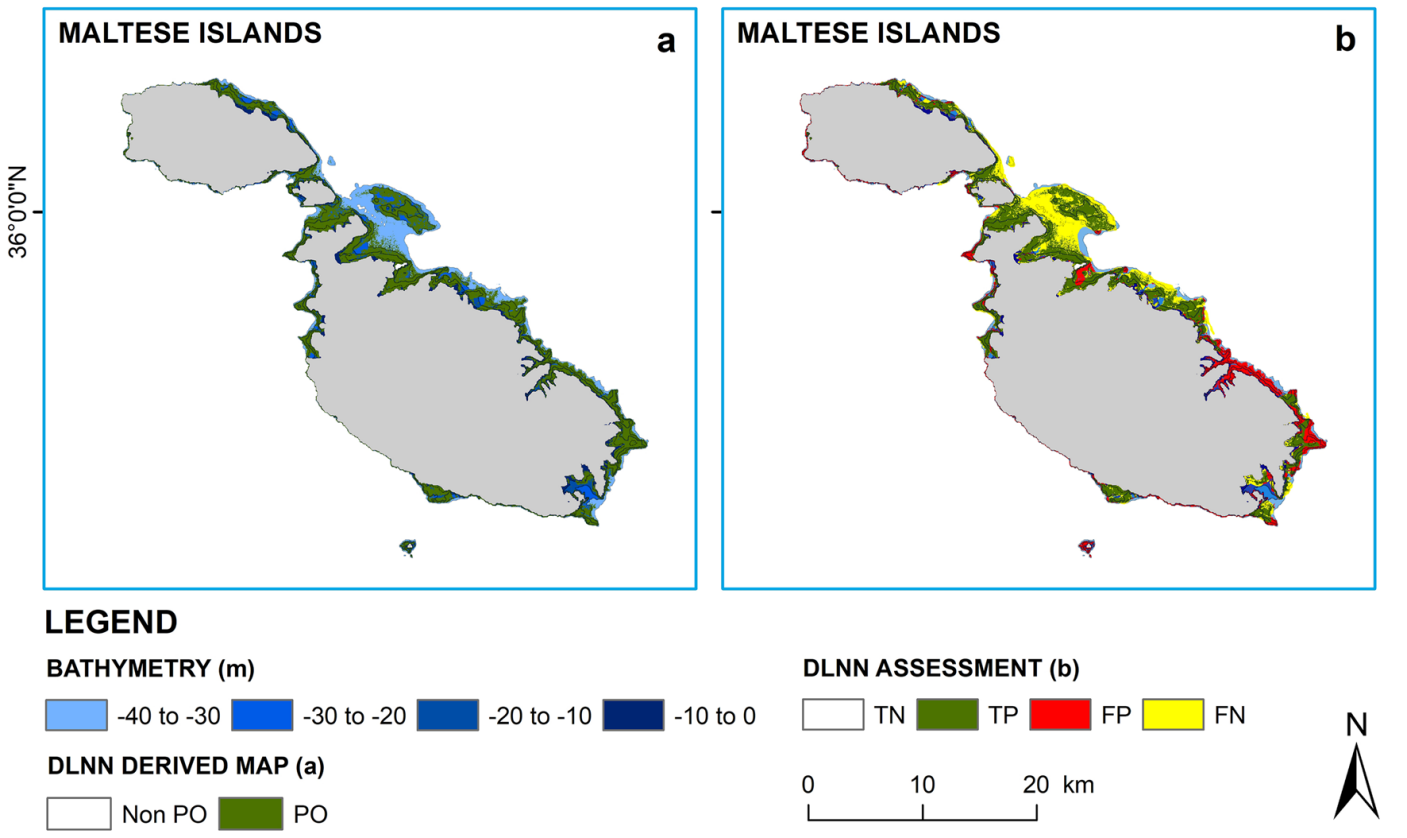
DLNN model trained with Formentera *in-situ* data. Map of *P. oceanica* in the Maltese Islands using the training dataset from Formentera. The maps have 10 m spatial resolution. Each pixel was identified as either PO or Non PO by the DLNN model. Subplot (**a**) demonstrates the spatial extent of the meadows identified by the DLNN model, whereas subplot (**b**) demonstrates the model’s performance per pixel for the Islands. TN is True Negatives (pixels correctly identified as Non-PO), TP is True Positives (pixels correctly identified as PO), FP is False Positives (Non-PO pixels classified as PO), and FN is False Negatives (PO pixels classified as Non-PO). The land is shown in grey colour. Python 3.7 (https://www.python.org/downloads/release/python-370/) and ArcGIS 10.5 (https://desktop.arcgis.com/en/arcmap/10.5/get-started/installation-guide/installing-on-your-computer.htm?).

One of the advantages of our proposed method is the classification scheme. We trained the DLNN model to learn how the colour of the sea bottom with and without *P. oceanica* varied with depth, thus included the effect of water column implicitly. Precisely, instead of correcting the data for water column absorption, we implicitly include it in the forward modelling of the inversion problem for the numerical stability in the large depth, unlike the available literature^39^ that corrected water column absorption and scattering explicitly for a depth limit of 21 m. Besides, we designed the DLNN model for pixel-by-pixel classification per tile. As a result, our model was easily adaptable to other regions. We obtained accurate and reliable results while training the DLNN model with *in-situ* data from the target region, however, without *in-situ* data, the model was still capable of producing reliable maps which could provide a first-hand overview. To apprehend this, we reproduced the map of *P. oceanica* in the Maltese Island using the training dataset from Formentera (Fig. 4). The final output showed an overall accuracy of 64% with a user’s accuracy of 73% and a producer’s accuracy of 69%. As expected, the model performance was lower than what we observed in Table 1, however, it was still scientifically acceptable and could provide a general idea of the spatial extent of the meadows. It was evident that in both cases (Figs. 3i,j, 4) the model identified *P. oceanica* in the southeastern part of Malta, implying strong evidence of the presence of the seagrass meadows.

### Yearly monitoring of *P. oceanica* and its change assessment

**Figure 5:**
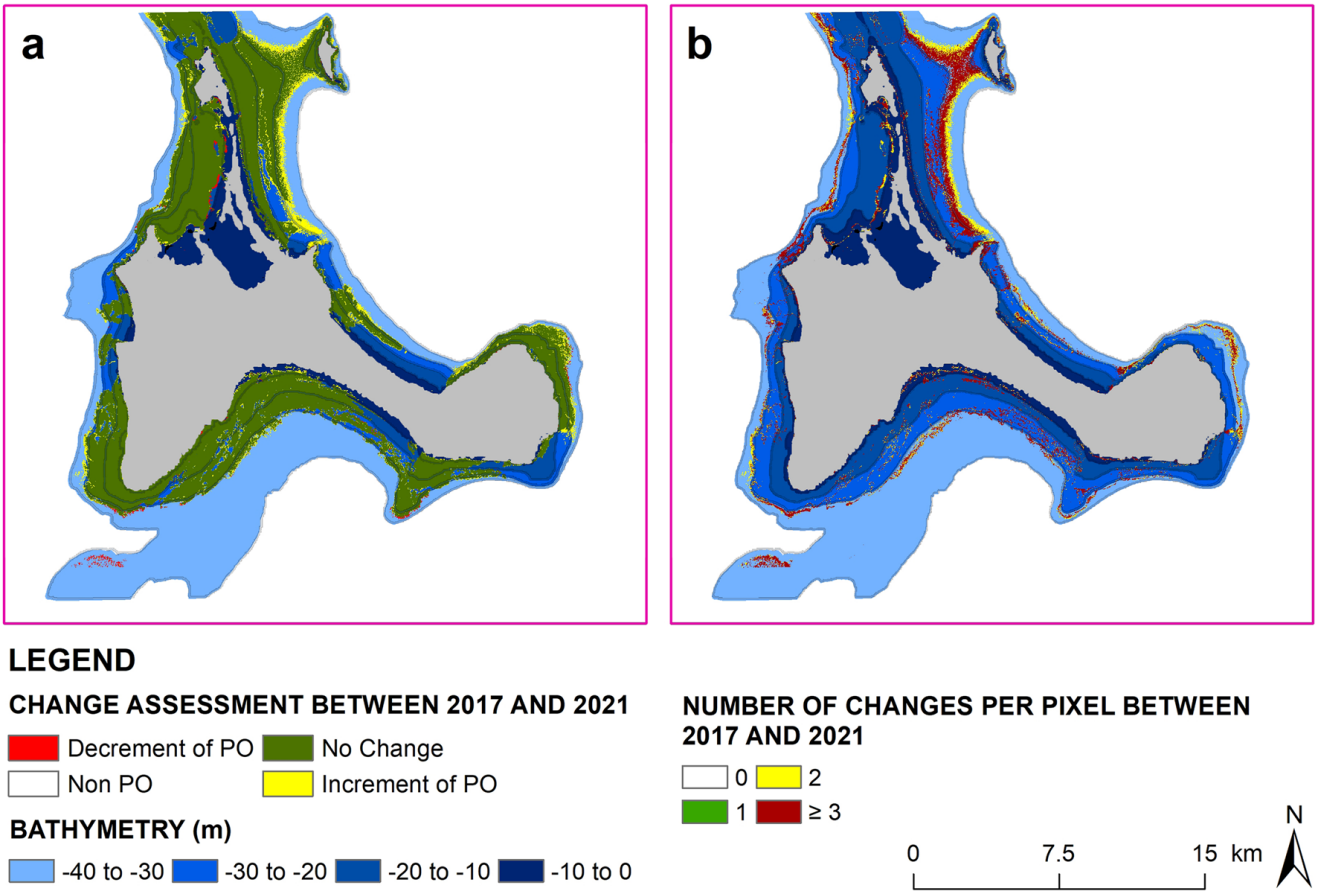
Change assessment of *P. oceanica* meadows in Formentera island during 2017–2021. Subplot (**a**) demonstrates the changes between the year of 2017 and 2021, where the green, yellow and red colours represent no change, increment and decrement of the *P. oceanica* meadows in 2021 compared to 2017. Subplot (**b**) demonstrates the number of changes per pixels from one class to another during this 5 years of period. Number 0 means the pixels were always *P. oceanica*, and the rest of the numbers (i.e., 1, 2 and ≥ 3) corresponded to the changes of the pixel from one class to another over the 5-year. The later was mostly observed at the optical depth limit (≥ 30 m depth) of the satellite where the model showed less accuracies compared to 0-25 m depth. Python 3.7 (https://www.python.org/downloads/release/python-370/) and ArcGIS 10.5 (https://desktop.arcgis.com/en/arcmap/10.5/get-started/installation-guide/installing-on-your-computer.htm?).

We applied our proposed approach to perform a time-series analysis (Fig. 5 and Supplementary Fig. S3, and Supplementary Table S2) in Formentera, as this region was highly renowned for conservation and restoration activities. While the seagrass patches mostly looked stable during 2017–2021 (‘0’ changes per pixel in Fig. 5b), we detected changes in the northern and northeastern parts of Formentera. Especially, in the northern part of the island at a depth of ≥ 30 m, we detected a loss of *P. oceanica* meadows until 2019 and then a subsequent gain since 2020 (Supplementary Fig. S3). It should be noted that during 2018 and 2019, the quality control for stacking was challenging due to poor quality of the available satellite images, which might results in the above-mentioned change detection at the optical depth limit for Sentinel-2. However, the change assessment between 2017 and 2021 (Fig. 5a) showed that the area of the meadow in the Formentera island increased by about 8 square km and decreased by about 1 square km, with a net gain of 7 square km, which might be a result of the current conservation and restoration activities. This showed the promising capabilities of our proposed approach to support monitoring of the meadows in the short and long term.

### Application in conservation and ecosystem management

In the recent past, the concept of natural recolonization (cutting and seedlings) has been introduced to restore *P. oceanica* meadows in the Mediterranean basin. However, most of them failed due to either choosing the wrong site where the species had never been before or a lack of long-term monitoring system due to economic constraints^43^. In addition, conservation and ecosystem management demand explicit and up-to-date knowledge to deal with real-time situations. Timely and reliable maps with a large spatial and temporal coverage can provide substantial ground in capacity building and making decisions. Unlike the available methodologies that were resource expensive^44^ and/or developed for a local scale^45^, our proposed method can generate maps of *P. oceanica* with adequate accuracy in a cost-effective manner. This can add value to the current restoration programmes by identifying suitable sites and monitoring them regularly for short-term goals and long-term evaluation. Besides, the information generated through our approach can support conservationists and ecosystem managers in making rational decisions to promote sustainability. This is particularly true for the MPN, which brings together different stakeholders such as, authorities, scientists, international environmental organizations, professionals including yachting agents and marinas from the Mediterranean countries. Being a part of the network, our approach can support the main objectives of this network, which is to protect all *P. oceanica* meadows in the Mediterranean Sea by 2030, and to increase each country’s capacity to protect these meadows by providing the necessary tools to prevent its future degradation.

**Figure 6:**
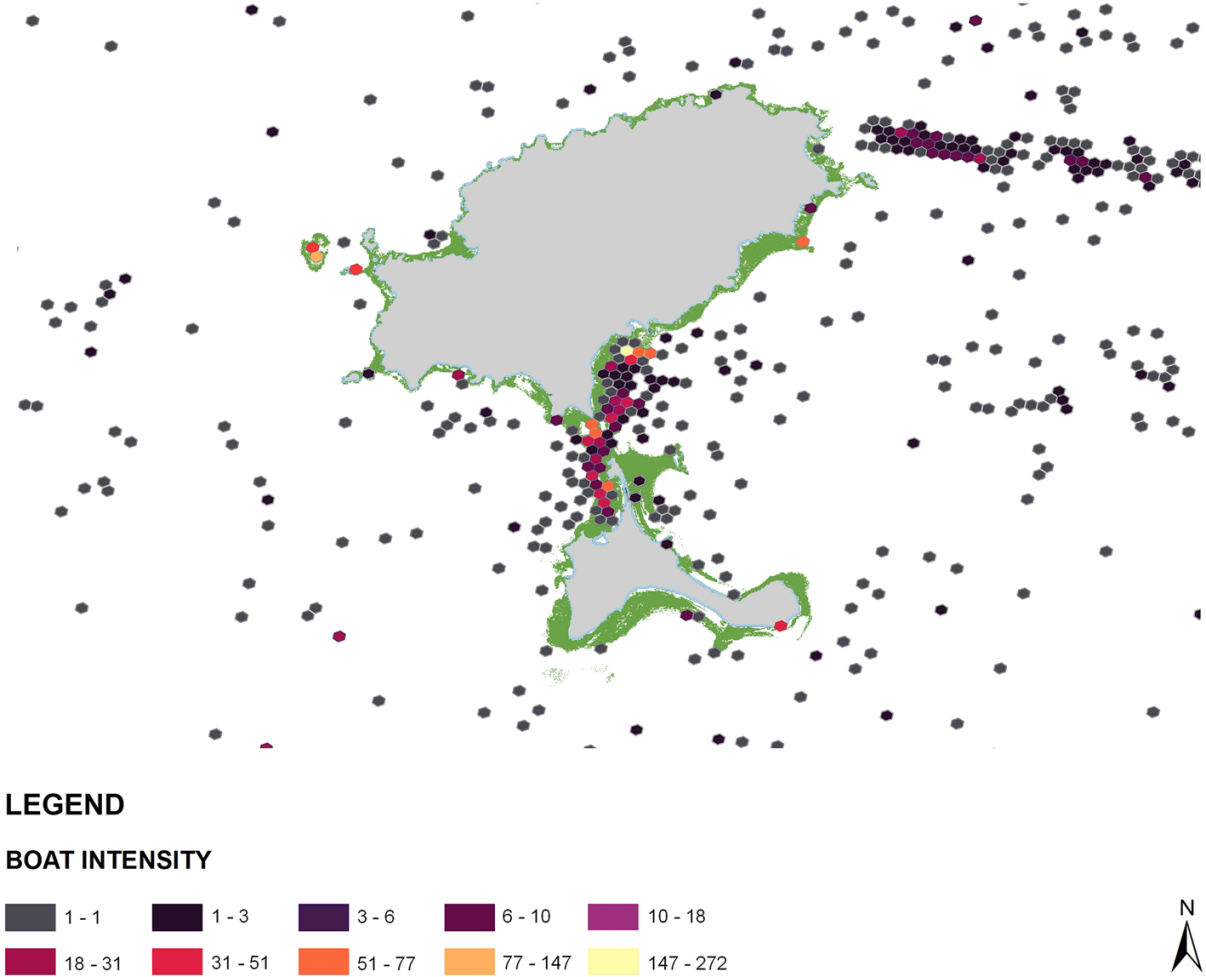
Boat intensity over the *P. oceanica* meadows. Boat pressure over the meadows in Formentera and Ibiza during June–August 2021, a peak tourist season. Each hexagon mesh presents the number of ships detected through Sentinel-1 satellite. Python 3.7 (https://www.python.org/downloads/release/python-370/) and ArcGIS 10.5 (https://desktop.arcgis.com/en/arcmap/10.5/get-started/installation-guide/installing-on-your-computer.htm?).

In this context, we present an example in Fig. 6. The figure demonstrates boat pressure over the seagrass patches in Formentera and Ibiza during June–August 2021, a peak tourist season. Each hexagon mesh presents the number of ships detected using Sentinel-1 satellite data. It was evident that the channel between Formentera and Ibiza had the highest pressure within this period, thus showing the promising capabilities to identify the hotspots of *P. oceanica* for conservation and sustainable coastal resource management.

This study facilitates the continuous and long-term monitoring of *P. oceanica* in the Mediterranean Sea in a fast and efficient manner by utilizing freely available high-resolution Sentinel-2 satellite imagery, low-resolution bathymetry data and open source free software (Python, Docker, etc.) to run the whole process automatically with minimal human interaction. Alongside the data-enriched regions, our approach can be used in data-scarce region, with environmental and other constraints that make it impossible to collect in-situ data, thus providing a first-hand overview. Besides, for precise mapping, our approach can be adaptable to high-resolution data, i.e., high-resolution bathymetry or commercial satellite data with RGB bands. Finally, our approach can be a stepping-stone for recording the long-term impact of climate change as well as different environmental drivers and stressors on the seagrass meadows following a standardized and uniform method, and ameliorate understanding of the connectivity between oceanography and seagrass ecosystem, spatial and temporal management and protection, and finally mapping other benthic ecosystems for conservation and management.

## Methods

### Scientific exploitation platform

To map *P. oceanica* in a fast, reliable and efficient way, we developed an operational pipeline (Supplementary Fig. S1) within a SEP. SEP integrated the hardware/software infrastructures that supplied the computing and storage resources needed for the exploitation and provision of the tools to manage EO datasets in a distributed environment. The platform comprised a Kubernetes cluster with many nodes hosted on-premises. The pipeline consisted of several scientific modules, such as data acquisition, data pre-processing, followed by artificial intelligence-based DLNN, and post-processing. Within the SEP, all these modules could be executed isolated or sequentially within Docker containers, encapsulating the entire environment required for the scientific workflow. This ensured scalability and the pipeline’s results could easily be reproduced with consistent outcomes. The pipeline also integrated a quality control system where a Network-file-system (NFS) volume held the raw data and the processed products, which could later be downloaded and analysed within the SEP web interface.

### Data acquisition

Sentinel-2 Level-1C data were downloaded from Google Cloud Storage using their Application Programming Interface (API). This data corresponded to the top of the atmospheric reflectance (TOA) for 7 Sentinel-2 tiles covering the Balearic Islands, and 1 tile covering the Maltese Islands (Supplementary Table S1). After visual inspection, images with high cloud coverage or sun glint effects were discarded from further processing. While mapping *P. oceanica*, bathymetry data plays a significant role, as this species is highly depth-dependent. In this study, the freely available bathymetry data at 100 m spatial resolution were obtained from the European Marine Observation and Data Network (EMODnet, https://www.emodnet-bathymetry.eu/). Besides, the in-situ data to develop and train the DLNN model were collected from the local government of the Balearic Islands^46^ (https://atlasposidonia.com/en/home/) and the Marine Database of Environment and Resources Authority (ERA) in Malta^47,48^. These data corresponded to single cartography in the GIS layer combining several years of field campaigns. In the case of Balearic Islands, the in-situ cartography were prepared based on the photointerpretation of aerial photographs between 0 and 5 m depth, side scan sonar campaign between 0 and 35 m depth, reprocessing of the information available from the LIFE *Posidonia* project, OCEANSNELL, Consell de Ibiza, Ecocartographies, etc. during 2008–2019^46,49^. These data corresponded to different marine habitats following the nomenclature and coding of the Standard List of Marine Habitats of Spain (LPHME). For the Maltese Islands, the in-situ cartography corresponded to the year of 2017–2019, having the information about the habitat of *P. oceanica.*

### Data pre-processing

Pre-processing is a crucial step while producing benthic maps using satellite data for artificial intelligence. It is even more important while using Sentinel-2 data for aquatic applications as it has low-signal-to-noise-ratio that requires a good atmospheric correction (AC) to separate the top-of-atmospheric (TOA) reflectance observed by the satellite into the signal from the atmosphere and the signal from the surface (bottom-of-atmosphere, BOA)^50^. In this study, we applied the ACOLITE (v2020) AC processor developed by the Royal Belgian Institute of Natural Sciences (RBINS). Based on the literature and the analysis results, we chose ACOLITE because it better reproduced the shape of the reflectance spectra than the other widely used AC processors, i.e., C2RCC (a SNAP plugin). As ACOLITE processor is image-based, it does not require any in-situ measurements. By default, ACOLITE performs AC using the ‘dark spectrum fitting’ (DSF) algorithm for aerosol corrections. However, this algorithm avoids severe glints for which the glint effects can still be found in the DSF-derived surface reflectance^51^. As sun glint was a prominent optical phenomenon in the study region, especially during spring and summertime, the optional glint correction within ACOLITE was also applied. The land and clouds were masked during the AC processing to retain the water-leaving reflectance. Therefore, the data were inspected visually. In some cases where high clouds (i.e. cirrus, cirrostratus, and cirrocumulus) and haze were left unmasked, and when these were found to degrade the region of interest near the coast, the images were discarded. Occasionally, images with high turbidity caused by river/land discharges or algae bloom were also discarded as they significantly affected the colour of the water and made it impossible to observe through the water column. These strict criteria only admitted images that were of very good quality. Therefore, to remove boats, very large waves, and other anomalies, as well as to reduce noisy patterns and artifacts in order to improve the overall quality of the image, a pixel-by-pixel median stacking was performed for the RGB bands of all the good quality atmospherically processed data (3–9 images per year). In this case, we chose median stacking as it was less affected by outliers and other anomalies compared to mean stacking, by preserving the features present in the majority of the images by taking the median value of each pixels across all the good quality images. In this way, as *P. oceanica* is a slow-growing species (1–6 cm/year of horizontal growth), the chances of overestimation of the seagrass extent because of the growth was negligible. Besides, if there was a sudden destruction of the meadows due to anthropogenic activities, it would likely lead to a drastic reduction in seagrass cover, resulting in a lower median value, thus reflecting the actual change more accurately.

The EMODnet bathymetry data was reprojected and resampled to 10 m (using the bilinear interpolation technique) to match the projection system and the spatial resolution of the Sentinel-2 data. The data was cropped after 40 m depth to remove open and deep ocean pixels to avoid potential FP of *P. oceanica*, as both classes had similar spectral values^37^ (Supplementary Fig. S2).

The marine habitats of the in-situ data were classified into two classes, i.e. "PO" (indicating the habitat of *P. oceanica*) and "Non-PO" (indicating other than the habitat of *P. oceanica*). *In-situ* data corresponding to land, offshore, and shallow water with a sandy/rocky habitat or other seagrass habitats were included in the Non-PO class. The PO and Non-PO classes were labelled as 1 and 0, respectively, and rasterized using the same projection system of Sentinel-2 scenes.

### Artificial intelligence-based DLNN approach

The identification of *P. oceanica* is a binary classification problem where pixels correspond to either PO or Non-PO. Therefore, the aim of using a DLNN model was to train a function based on the given satellite and bathymetry dataset (X) as well as target in-situ data (Y) to output a predicted class (Ŷ) of PO and Non-PO, which should match as closely as possible with the in-situ data (Y). In this study, we have randomly split the data into 80/20 ratios per Sentinel-2 tile, where 80% data were used for training and the rest 20% were used for validation of the model.

The training was done through two processes, namely, forward propagation and backpropagation to recognize patterns based on the given dataset. In forward propagation, the process moved in the forward direction through the neural network (NN) to produce a final value at the output layer, whereas at the same time, in backpropagation, the process moved from the output layer to the input layer to improve the weight (w) value generated in the forward process. Forward propagation was a value-processing step from the input layer that involved calculating a linear combination per neuron using adjustable linear coefficients and then calculating an activation function by performing a non-linear transformation for the corresponding neuron within the layers. The activation functions of all the layers except the last one were the rectified linear unit activations (ReLU), meaning that the output was set to zero if it was negative. Hence, the output coefficients from the neurons never had negative values. In the DLNN model, the last layer was a binary classifier, which means that it classified pixels as belonging to either class 1 or class 0. In this case, the activation function was a sigmoid function, which predicted the probability (Ŷ) of a pixel being class 1 or 0 based on a threshold value of 0.5.

To optimize the training process, we performed mini-batch processing, where we split the training set into smaller sets and implemented gradient descent on each batch chronologically to make the algorithm work faster. Therefore, based on the input data, each 10 m pixel of the Sentinel-2 image was classified as containing PO (class 1) or Non-PO (class 0). The result, Ŷ, was compared to Y, and the difference was quantified in a cost function. Minimizing the cost function equates to making Ŷ as similar as possible to Y. The differences between Ŷ and Y per pixel allowed the calculation of the gradient that quantified how much each coefficient in each neuron must be altered to match Ŷ to Y. This gradient was then used to update the coefficients in the neurons of all the layers through backpropagation. This was an iterative process, and therefore, at each iteration, a new prediction was made, compared to the target Y, a gradient was calculated, and the coefficients were updated. This process was carried out continuously until it produced the smallest loss value, and the output was saved in the form of a file containing the weight (w) and bias (b) values. Afterward, the derived set of coefficients from the DLNN model was used to map *P. oceanica*.

### Post-processing and accuracy assessment

In the post-processing step, we evaluated several filters, i.e. sieve (replacing small isolated groups of pixels with the pixel value of the largest neighbour), median (ranking values of the pixels in the moving window with a specified radius and taking the middle-ranking value), bilateral (replacing the value of each pixel with a weighted average of the pixel values in the moving window), and principal component analysis (removing noise by discarding the last principal components that explain the least of variance). Among them, we chose the median filter with 3 × 3 pixels moving window as it significantly reduced the noise while preserving the shape of the seagrass patches. We applied the filter to the binary maps and merged the resultant maps per region to produce the regional map. Then we calculated the overall, producer’s, and user’s accuracies of the final map using the corresponding geo-referenced in-situ validation dataset. The overall accuracy expressed the ratio of the number of correctly classified pixels to the total number of given pixels regardless of the class. Producer’s accuracy expressed how often *P. oceanica* could be identified correctly on the classified map; whereas, the user’s accuracy expressed how often the *P. oceanica* class on the map would be present on the ground. Producer’s accuracy is the map accuracy from the mapmaker’s viewpoint and so it is a great statistical metric for the remote sensing scientist creating the habitat map. In contrast, the user’s accuracy is the accuracy from the map user’s viewpoint. Hence, it is more significant in a management context of a given region as it reports a quantitative probability for the actual presence of the studied habitat (in this case, *P. oceanica*) in the given region^38^. The equations for all the aforementioned classification metrics are given below:

1 $$\mathrm{Overall \; accuracy }= \frac{TP+TN}{n}$$

2 $$\mathrm{Producer{\prime}}\mathrm{s \; accuracy }= \frac{TP}{TP+FN}$$

3 $$\mathrm{User{\prime}}\mathrm{s \; accuracy }= \frac{TP}{TP+FP}$$

Here, TP is the number of true positives, i.e., pixels correctly identified as PO; TN is the number of true negatives, i.e., pixels correctly identified as Non-PO; FP is the number of false positives, i.e., the number of actual Non-PO pixels classified as PO; and FN is the number of false negatives, i.e., the number of actual PO pixels classified as Non-PO.

### Supplementary Information

Supplementary Information.

## Data Availability

The necessary procedures to generate the data and reproduce the methodology have been outlined in the manuscript. The data that support the findings of this study are available from the authors on reasonable request.
