# Supplementary material for: AI-driven remote sensing enhances Mediterranean seagrass monitoring and conservation to combat climate change and anthropogenic impacts
Source: Sci Rep. 2024 Apr 10;14:8360. doi: 10.1038/s41598-024-59091-7 (PMC11006664; doi:10.1038/s41598-024-59091-7)
Supplement: Supplementary file 1 — Supplementary Information. [file 41598_2024_59091_MOESM1_ESM.pdf]

AI-driven remote sensing enhances Mediterranean seagrass monitoring and conservation to combat climate change and anthropogenic impacts

Masuma Chowdhury*[1,3], Alejo Martínez-Sansigre[1], Maruška Mole[1], Eduardo Alonso Peleato[1], Nadiia Basos[1], Jose Manuel Blanco[1], Maria Ramirez[1], Isabel Caballero[2], Ignacio de la Calle[1]

[1]Quasar Science Resources, S. L. Camino de las Ceudas 2, 28232 Las Rozas de Madrid,

Madrid, Spain

masuma.chowdhury@quasarsr.com, amartinez@quasarsr.com, maruska.mole@quasarsr.com, eduardo.alonso@quasarsr.com, nbasos@quasarsr.com, josemanuelblanco@quasarsr.com, maria.ramirez@quasarsr.com, ignaciodelacalle@quasarsr.com

[2]Instituto de Ciencias Marinas de Andalucía (ICMAN), Consejo Superior de

Investigaciones Científicas (CSIC), Avenida República Saharaui, 11510 Cadiz, Spain

isabel.caballero@icman.csic.es

[3]Departamento de Física Aplicada, Instituto Universitario de Investigación Marina (INMAR), Universidad de Cádiz, Campus de Excelencia Internacional/Global del Mar (CEI·MAR), Puerto Real (Cadiz), Spain

masuma.chowdhury@uca.es

*Corresponding author: masuma.chowdhury@quasarsr.com

Supplementary Table S1. Details of the satellite images used in Figure 3. The naming convention of the satellite data corresponds to satellite name, sensor name, data level, acquisition date, and tile and orbit names, respectively.

Southwestern Mallorca	S2B_MSI_L1C_2021_01_17_T31SDD_R008
	S2A_MSI_L1C_2021_03_23_T31SDD_R008
	S2B_MSI_L1C_2021_03_28_T31SDD_R008
	S2B_MSI_L1C_2021_09_04_T31SDD_R008
	S2A_MSI_L1C_2021_10_09_T31SDD_R008
Southeastern Mallorca	S2A_MSI_L1C_2021_06_08_T31SED_R108
	S2A_MSI_L1C_2021_07_18_T31SED_R108
	S2A_MSI_L1C_2021_07_28_T31SED_R108
	S2A_MSI_L1C_2021_08_07_T31SED_R108
	S2A_MSI_L1C_2021_08_27_T31SED_R108
Northern Mallorca	S2B_MSI_L1C_2021_01_17_T31TDE_R008
	S2A_MSI_L1C_2021_02_01_T31TDE_R008
	S2A_MSI_L1C_2021_02_11_T31TDE_R008
	S2A_MSI_L1C_2021_03_23_T31TDE_R008
	S2B_MSI_L1C_2021_03_28_T31TDE_R008
	S2A_MSI_L1C_2021_04_02_T31TDE_R008
	S2A_MSI_L1C_2021_10_09_T31TDE_R008
Northeastern Mallorca and Western Menorca	S2A_MSI_L1C_2021_05_29_T31TEE_R108
	S2A_MSI_L1C_2021_07_18_T31TEE_R108
	S2B_MSI_L1C_2021_07_23_T31TEE_R108
	S2B_MSI_L1C_2021_08_22_T31TEE_R108
	S2A_MSI_L1C_2021_08_27_T31TEE_R108
Eastern Menorca	S2B_MSI_L1C_2021_03_25_T31TFE_R108
	S2A_MSI_L1C_2021_03_30_T31TFE_R108
	S2A_MSI_L1C_2021_07_28_T31TFE_R108
	S2A_MSI_L1C_2021_03_10_T31TFE_R108
	S2A_MSI_L1C_2021_05_29_T31TFE_R108
	S2A_MSI_L1C_2021_07_18_T31TFE_R108
	S2B_MSI_L1C_2021_07_23_T31TFE_R108
Formentera	S2B_MSI_L1C_2021_01_17_T31SCC_R008
	S2A_MSI_L1C_2021_03_13_T31SCC_R008
	S2B_MSI_L1C_2021_05_07_T31SCC_R008
	S2B_MSI_L1C_2021_06_26_T31SCC_R008
	S2A_MSI_L1C_2021_07_01_T31SCC_R008
	S2A_MSI_L1C_2021_08_30_T31SCC_R008
	S2B_MSI_L1C_2021_10_24_T31SCC_R008
Ibiza	S2A_MSI_L1C_2021_01_12_T31SCD_R008
	S2A_MSI_L1C_2021_02_11_T31SCD_R008
	S2A_MSI_L1C_2021_03_13_T31SCD_R008
	S2A_MSI_L1C_2021_03_23_T31SCD_R008
	S2A_MSI_L1C_2021_05_02_T31SCD_R008
	S2A_MSI_L1C_2021_05_12_T31SCD_R008
	S2B_MSI_L1C_2021_07_16_T31SCD_R008
	S2B_MSI_L1C_2021_08_05_T31SCD_R008

	S2B_MSI_L1C_2021_10_24_T31SCD_R008
The Maltese Islands	S2A_MSI_L1C_2021_02_06_T33SVV_R079
	S2B_MSI_L1C_2021_04_02_T33SVV_R079
	S2A_MSI_L1C_2021_04_07_T33SVV_R079

Supplementary Table S2. Details of the satellite images used in Figures 5 and S3. The naming convention of the satellite data corresponds to satellite name, sensor name, data level, acquisition date, and tile and orbit names, respectively.

**Year**	**Satellite Image**
2021	S2B_MSI_L1C_2021_01_17_T31SCC_R008
	S2A_MSI_L1C_2021_03_13_T31SCC_R008
	S2B_MSI_L1C_2021_05_07_T31SCC_R008
	S2B_MSI_L1C_2021_06_26_T31SCC_R008
	S2A_MSI_L1C_2021_07_01_T31SCC_R008
	S2A_MSI_L1C_2021_08_30_T31SCC_R008
	S2B_MSI_L1C_2021_10_24_T31SCC_R008
2020	S2B_MSI_L1C_2020_01_13_T31SCC_R008
	S2A_MSI_L1C_2020_01_28_T31SCC_R008
	S2B_MSI_L1C_2020_02_02_T31SCC_R008
	S2B_MSI_L1C_2020_02_22_T31SCC_R008
	S2A_MSI_L1C_2020_04_07_T31SCC_R008
	S2B_MSI_L1C_2020_05_02_T31SCC_R008
	S2A_MSI_L1C_2020_05_27_T31SCC_R008
	S2B_MSI_L1C_2020_06_21_T31SCC_R008
	S2A_MSI_L1C_2020_06_26_T31SCC_R008
2019	S2B_MSI_L1C_2019_03_09_T31SCC_R008
	S2A_MSI_L1C_2019_03_14_T31SCC_R008
	S2A_MSI_L1C_2019_03_24_T31SCC_R008
	S2A_MSI_L1C_2019_04_13_T31SCC_R008
	S2B_MSI_L1C_2019_04_28_T31SCC_R008
	S2A_MSI_L1C_2019_05_23_T31SCC_R008
	S2B_MSI_L1C_2019_09_25_T31SCC_R008
2018	S2A_MSI_L1C_2018_04_18_T31SCC_R008
	S2B_MSI_L1C_2018_07_02_T31SCC_R008
	S2A_MSI_L1C_2018_07_17_T31SCC_R008
	S2B_MSI_L1C_2018_08_01_T31SCC_R008
	S2A_MSI_L1C_2018_08_06_T31SCC_R008
	S2B_MSI_L1C_2018_08_21_T31SCC_R008
	S2A_MSI_L1C_2018_11_04_T31SCC_R008
	S2B_MSI_L1C_2018_11_29_T31SCC_R008
2017	S2A_MSI_L1C_2017_05_03_T31SCC_R008
	S2A_MSI_L1C_2017_05_13_T31SCC_R008
	S2A_MSI_L1C_2017_05_23_T31SCC_R008
	S2A_MSI_L1C_2017_06_12_T31SCC_R008
	S2A_MSI_L1C_2017_06_22_T31SCC_R008
	S2B_MSI_L1C_2017_07_17_T31SCC_R008
	S2B_MSI_L1C_2017_10_25_T31SCC_R008

Supplementary Figure S1: Schematic workflow

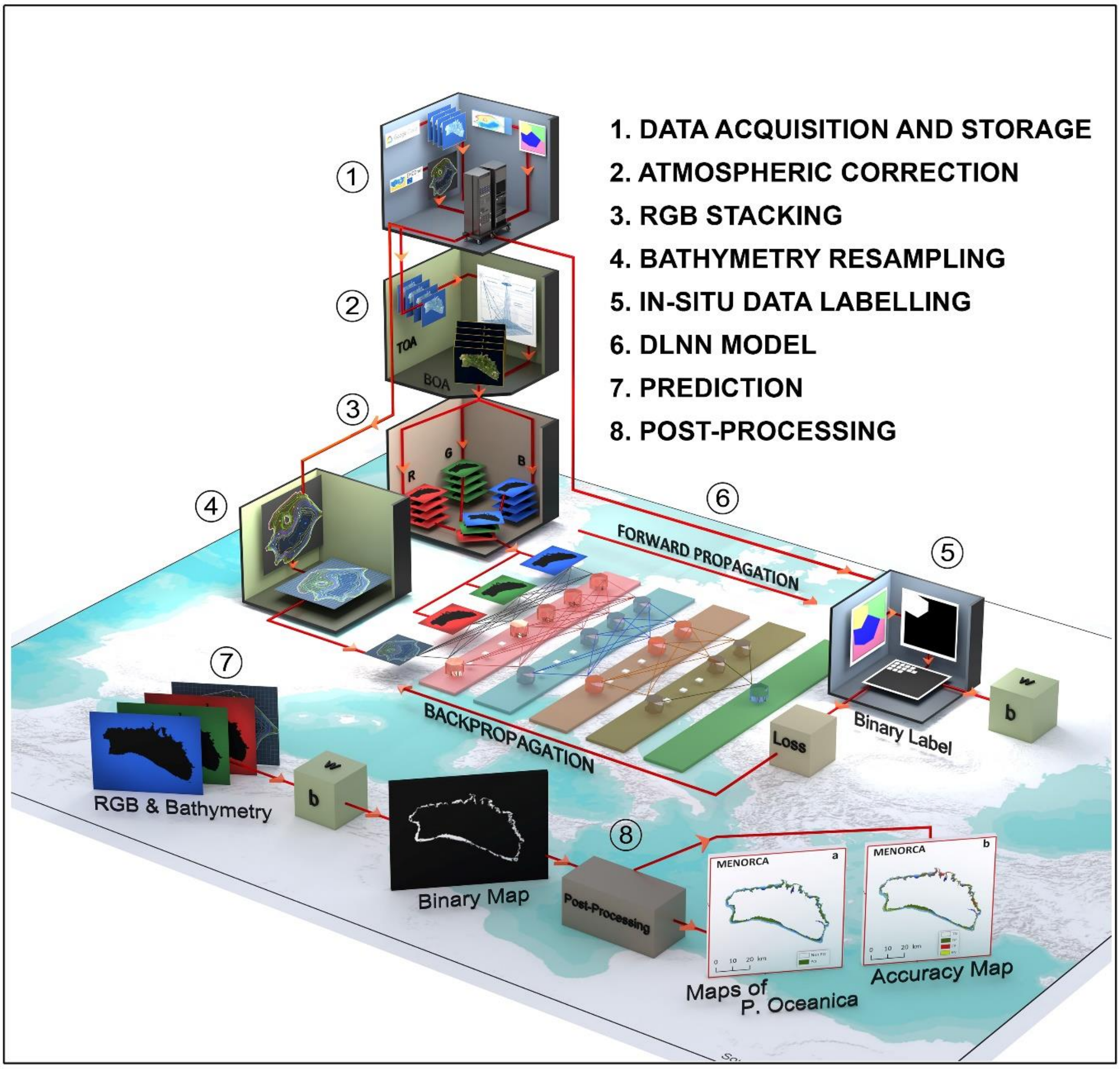

Schematic pipeline, starting with downloading satellite, bathymetry and in-situ data (1), applying atmospheric correction on Sentinel-2 Level-1C top of atmospheric (TOA) reflectance (2), stacking yearly atmospherically corrected red (R), green (G) and blue (B) bands (3), resampling bathymetry data (4), labelling and rasterizing in-situ data (5), training the DLNN model and output weight (w) and bias (b) (6), predicting the spatial extent of *P. oceanica* (7), and Post-processing to output final maps (8). The arrows indicate the successive steps. Sketchup 2022 (https://www.sketchup.com/offline-download) and Photoshop 2023 (https://www.adobe.com/products/photoshop.html)

Supplementary Figure S2: Bathymetry of the study regions

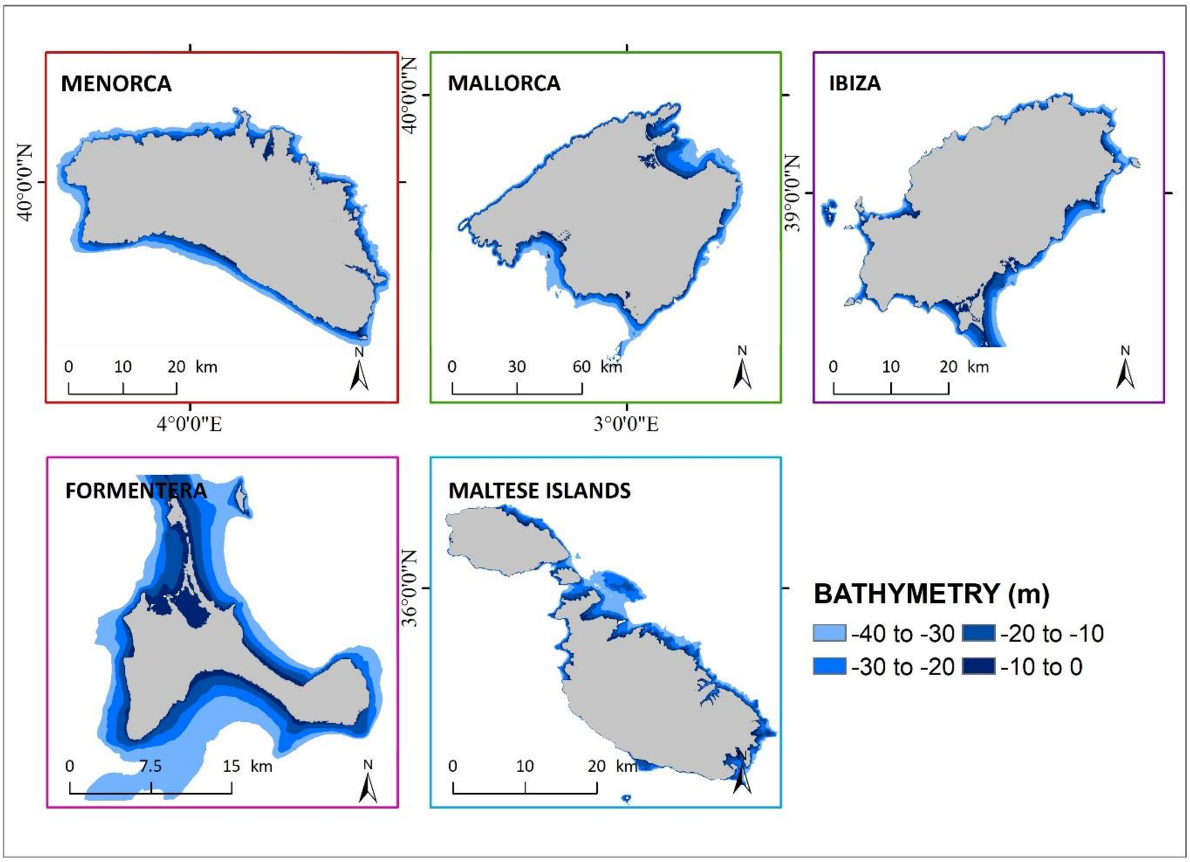

EMODnet bathymetry data used as an input of the DLNN model in mapping *P. oceanica* in the Balearic and the Maltese Islands in the Mediterranean Sea. This data was reprojected and resampled to 10m (using the bilinear interpolation technique) to match the projection system and the spatial resolution of the Sentinel-2 data. The data was cropped after 40m depth to remove open and deep ocean pixels to avoid potential missclassification. Python 3.7 (https://www.python.org/downloads/release/python-370/) and ArcGIS 10.5 (https://desktop.arcgis.com/en/arcmap/10.5/get-started/installation-guide/installing-on-your-computer.htm?)

Supplementary Figure S3: Yearly maps of *P. oceanica* meadows during 2017-2021

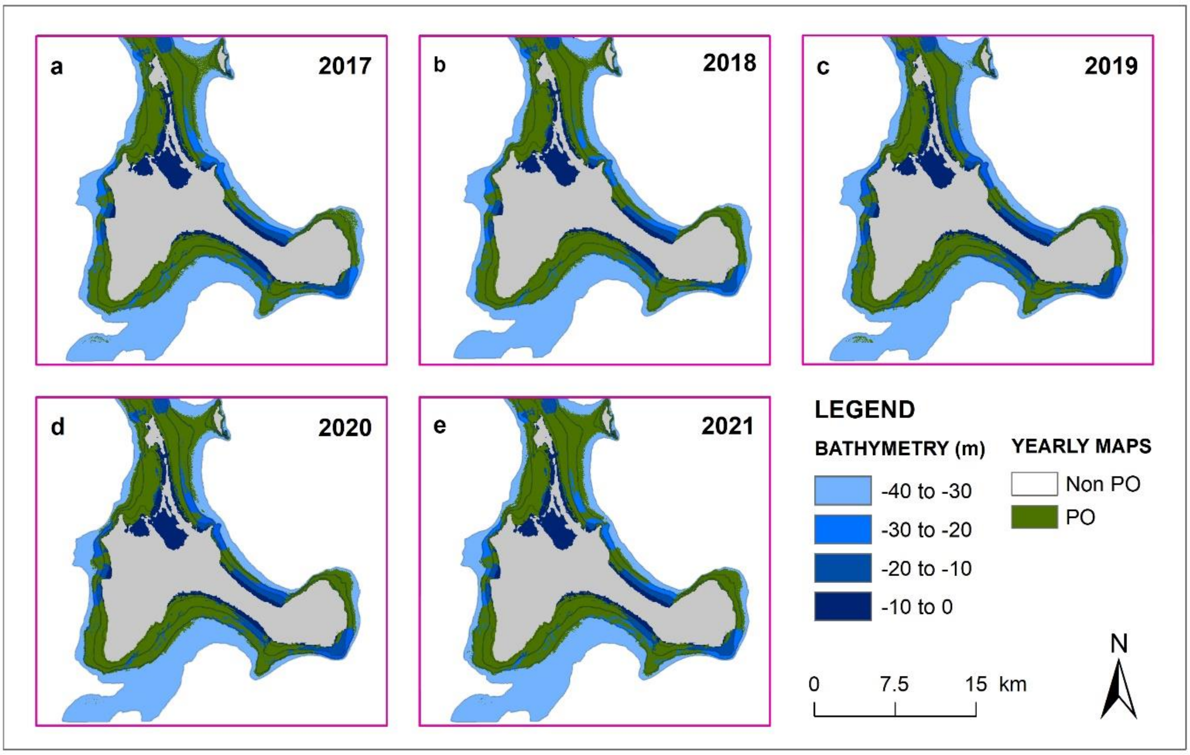

Yearly maps of *P. oceanica* in Formentera during 2017-2021. The seagrass patches mostly looked stable during this period, except the northern part of the Island (at the depth limit of satellite data, i.e., ≥30m), where we detected a loss of the meadows until 2019 and then a subsequent gain since 2020, which could be a result of the poor quality of the satellite images (that caused residual noise in the stacked images) used in mapping the meadows. Python 3.7 (https://www.python.org/downloads/release/python-370/) and ArcGIS 10.5 (https://desktop.arcgis.com/en/arcmap/10.5/get-started/installation-guide/installing-on-your-computer.htm?)
